# Primary diffuse leptomeningeal atypical teratoid/rhabdoid tumours (ATRT) of childhood: a molecularly characterised case report and literature review

**DOI:** 10.1007/s00381-024-06698-w

**Published:** 2025-01-22

**Authors:** S. M. Stivaros, L. M. Parkes, R. Bedir, E. Cheesman, D. Ram, L. Leung, A. Huang, J. P. Kilday

**Affiliations:** 1 The Geoffrey Jefferson Brain Research Centre, University of Manchester, Manchester Academic Health Science Centre, Manchester, UK; 2 Division of Informatics, Imaging and Data Sciences, School of Health Sciences, Faculty of Biology, Medicine and Health, University of Manchester, Manchester Academic Health Science Centre, Manchester, UK; 3 School of Health Sciences Faculty of Biology Medicine and Health University of Manchester, Manchester, UK; 4 Children’s Brain Tumour Research Network (CBTRN), Royal Manchester Children’s Hospital, Oxford Road, Manchester University NHS Foundation Trust, Manchester, UK; 5 Department of Histopathology, Royal Manchester Children’s Hospital, Oxford Road, Manchester University Hospitals NHS Foundation Trust, Manchester, UK; 6 Arthur and Sonia Labatt Brain Tumour Research Centre, The Hospital for Sick Children, Toronto, Canada; 7 Department of Laboratory Medicine and Pathobiology, The Hospital for Sick Children, Toronto, Canada; 8 The Centre for Paediatric Teenage and Young Adult Cancer, Institute of Cancer Sciences, The University of Manchester, Manchester, UK

**Keywords:** Paediatric diffuse leptomeningeal, ATRT, ASL, Craniospinal radiotherapy

## Abstract

**Background:**

Atypical teratoid/rhabdoid tumours (ATRTs) are malignant central nervous system tumours, typically presenting in the posterior fossa of very young children. Prognosis remains poor despite current therapy, while tumorigenesis implicates both genomic and epigenetic dysregulation. Primary diffuse leptomeningeal (PDL) ATRT, characterised by the absence of an intraparenchymal mass lesion, is seldom reported but appears associated with a dismal outcome.

**Case presentation:**

We describe a 7-year-old male presenting with a PDL MYC-subgroup ATRT. The patient received multimodal upfront therapy, including high-dose craniospinal radiotherapy, embedded within a chemotherapy backbone. An unexpected clinical and radiological improvement was also observed upon cessation of all therapy for presumed disease progression. Although the patient eventually succumbed to the disease at 30 months, he demonstrated the longest survival for any PDL ATRT patient reported (median 8 months).

**Conclusion:**

Exhaustive literature review identified seven preceding published cases of PDL ATRT. Ours is the only one to have molecular subgrouping assigned. Perfusion imaging, within a multi-parametric diagnostic package, may be a sensitive marker for malignancy against other aetiologies in challenging presentations. Acknowledging the scarcity of the entity, we cautiously suggest a combination of chemotherapy and upfront high-dose craniospinal radiotherapy, if appropriate, may prolong survival for older children with PDL ATRT compared to exclusive chemotherapy or focal irradiation-based strategies. Our patient’s recovery during palliation following a radiological diagnosis of disseminated relapse highlights the importance of confirming disease recurrence by tissue extraction where feasible.

**Supplementary Information:**

The online version contains supplementary material available at 10.1007/s00381-024-06698-w.

## Introduction

Atypical teratoid/rhabdoid tumours (ATRTs) are malignant embryonal tumours of the central nervous system (CNS), typically arising in very young children [1]. A posterior fossa location is prevalent in those aged below 2 years, while supratentorial ATRTs are often seen in the older age group [2–4]. Surgery and adjuvant therapies are informed by factors including patient age, tumour location and stage [5], yet universal agreement on optimal therapeutic strategies remains elusive. Consequently, ATRTs remain a clinical challenge with a predominantly poor prognosis.

Almost all ATRTs are molecularly characterised by the inactivation of the chromatin-regulating tumour suppressor gene *SMARCB1* [6]. Despite this relative genomic homogeneity, ATRTs can demonstrate marked clinico-biological disparity. Indeed, recent consensus has now confirmed three clinically, genetically and epigenetically unique molecular ATRT subgroups: TYR, SHH and MYC [4].

While metastatic dissemination in ATRT is well reported [2], primary diffuse leptomeningeal (PDL) infiltration, without an intraparenchymal CNS mass lesion, is exceptionally rare. Only seven cases have been published in global literature (Table 1, [7–12]); only four represent children below 16 years of age [8, 9, 12]. Molecular subgroup evaluation has never been documented.

**Table 1:** Published cases of primary diffuse leptomeningeal ATRT in the paediatric, teenage and young adult age group

Study	Age (years)	Gender	Location (on MRI)	Primary treatment	Genetic analysis	Disease status	Length of follow-up
Present case	7	Male	Right cerebral hemisphere LMD	EU-RHAB chemotherapy (pre-RT intraventricular methotrexate, 9 cycles of ICE (× 3), VCA (× 3) and doxorubicin (× 3)) and CSRT (35.2 Gy in 22 fractions) + 19.8 Gy in 11 fractions (cumulative 55 Gy) to disease sites in the right cerebral hemisphere	a) *SMARCB1* deletion upstream of exon 9 b) Larger (approx. 10 Mb) chromosome 22 deletion including *SMARCB1* c) ATRT-MYC molecular subgroup	Spinal relapse 3 months from diagnosis. DOD 30 months from diagnosis (12 months from palliation)	30 months from diagnosis
Kayo et al. (2022) [10]	16	Female	Right frontal lobe nodule with LMD in bilateral cerebral/cerebellar hemispheres and spine	Induction chemotherapy (methotrexate, vincristine, cyclophosphamide and etoposide)	Not documented	Not documented but therapy abandoned after 6 days due to status epilepticus	Not documented
Tomomasa et al. (2018) [12]	15	Male	LMD in bilateral cerebral/cerebellar hemispheres and brainstem	Modified IRS-III protocol, accompanied by radiotherapy (36 Gy for the whole brain)	a) FISH heterozygous loss of the *hSNF5/INI1* region (22q11.23) b) MLPA showed heterozygous microdeletion (exons 1–5 on 1 allele, exons 1–7 in other allele) c) Mutational sequencing analysis negative	8 months from diagnosis	8 months from diagnosis
Baute et al. (2017) [7]	25	Male	Intracranial and spinal LMD	RT (36 Gy in 24 fractions) to spinal disease at T2–T5 and L3–L4 followed by adjuvant chemotherapy (not specified)	FISH loss of the *hSNF5/INI1* region (22q11.23)	DOD 12 months from diagnosis	12 months from diagnosis
Livermore et al. (2013) [11]	17	Male	Enhanced left glossopharyngeal nerve, bilateral trigeminal nerves, LMD distal spinal cord	EU-RHAB chemotherapy (pre-RT intraventricular methotrexate, 6 cycles of ICE (× 2), VCA (× 2) and doxorubicin (× 2)) and CSRT (dose specifics not documented)	Not documented	DOD 25 months from diagnosis	25 months from diagnosis
Gauvain et al. (2012) [9]	1.3	Male	Enhanced oculomotor nerve, medullary expansion with nodule and LMN around 4th ventricle & hydrocephalus, spinal mass L1-L2	Nil as DOD before treatment initiated—diagnosis made post-mortem	Not documented	6–8 weeks from presentation	6–8 weeks from presentation
Gauvain et al. (2012) [9]	2.3	Male	LMD, mostly in quadrigeminal plate and posterior fossa, upper spinal cord	Nil as decision to withdraw care due to clinical status—DOD	Not documented	6 weeks from presentation	6 weeks from presentation
El-Nabbout et al. (2010) [8]	2.5	Female	Intracranial and spinal LMD	Nil as DOD before treatment initiated	FISH loss of the *hSNF5/INI1* region (22q11.23) in 100% of interphase cells	Not documented but weeks from presentation	Not documented but weeks from presentation

Key: *MRI*, magnetic resonance imaging; *LMD*, leptomeningeal disease; EU-RHAB protocol [13]; IRS-III protocol [27]; *ICE*, ifosfamide; carboplatin and etoposide; *Dox*, doxorubicin; *VCA*, vincristine; cyclophosphamide; actinomycin; *CSRT*, craniospinal radiotherapy; *Mb*, megabase; *EOT*, end of therapy; *DOD*, died of disease

We resolve this by presenting a novel case of MYC-subgroup PDL ATRT, demonstrating the longest overall survival reported when compared with the published cohort. The patient’s clinical trajectory included an unanticipated radiological and clinical improvement from a radiological diagnosis of disease recurrence without conventional therapies, explanations for which are offered.

## Case report

**Fig. 1:**
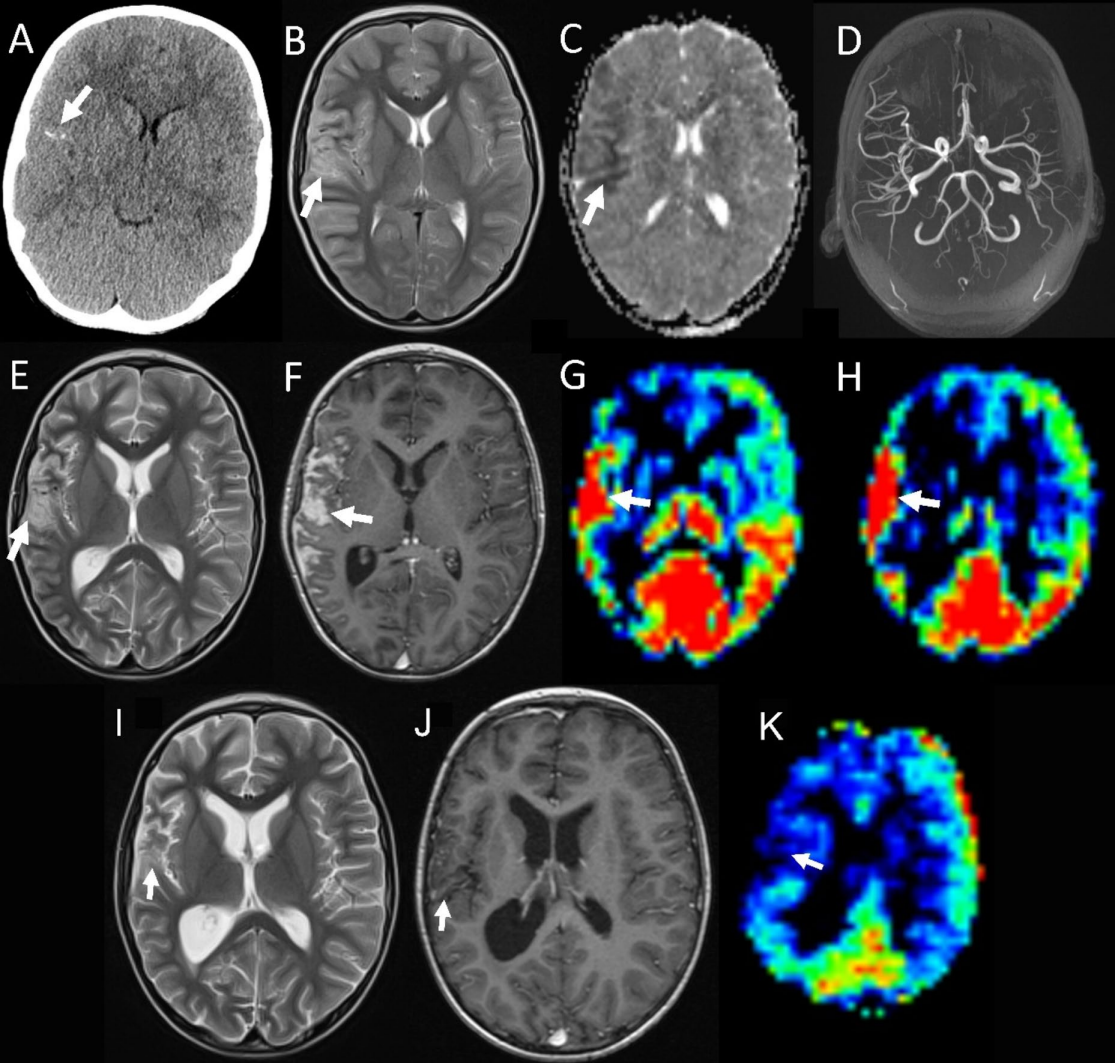
Presentation and primary treatment imaging. Non-contrast CT demonstrated what was initially thought to represent a right MCA thrombus occlusion (panel A arrow). MR imaging 1 day later demonstrated increased oedema on axial T2 sequences and associated diffusion restriction in an MCA distribution on the ADC map (panels B and C respectively, arrows). Yet, no major vessel occlusion was seen on the time-of-flight MRA (panel D). Panels E–H are taken from the re-presentation MRI scan some 5 weeks later. Axial T2 sequences showed worsening oedema (panel E, arrow). The T1 post-contrast image (panel F) showed significant leptomeningeal thickening and enhancement (white arrow) but no mass lesion. Concurrent ASL demonstrated significantly increased perfusion mapping to the leptomeningeal disease (panels G and H, arrows). By the end of therapy, the axial T2 (panel I) showed a reduction in peri-sylvian oedema (arrow) with a resolution to the previously seen contrast enhancement (panel J, arrow). This area also demonstrated post-treatment volume loss. The ASL sequence shows resolution to the previously seen increased perfusion (panel K, arrow, versus G and H; see Supplementary Information)

An otherwise healthy 7-year-old male presented acutely with left-sided focal seizures followed by secondary generalisation. CT brain imaging demonstrated what was thought to be a right middle cerebral artery (MCA) infarction, with non-contrast MRI showing corresponding right MCA territory oedema and diffusion restriction. However, MR angiography failed to identify the occlusive disease (Fig. 1A–D). Inflammatory and metabolic vasculopathy mimics were excluded, while echocardiography was normal. The patient improved within a week to discharge following infective encephalitis therapy and combined levetiracetam and phenytoin. He re-presented 5 weeks later with acute headache, vomiting, left hemiparesis and collapse. Contrast-enhanced neuraxial MR imaging revealed leptomeningeal infiltration of the right frontal, parietal and temporal lobes with corresponding diffusion restriction and increased perfusion on arterial spin labelling (ASL) imaging. There was no discernible mass lesion (Fig. 1E–H). Given the leading diagnosis was now of a malignancy, a right frontal brain biopsy was performed, incorporating gyral and sulcal surfaces.

**Fig. 2:**
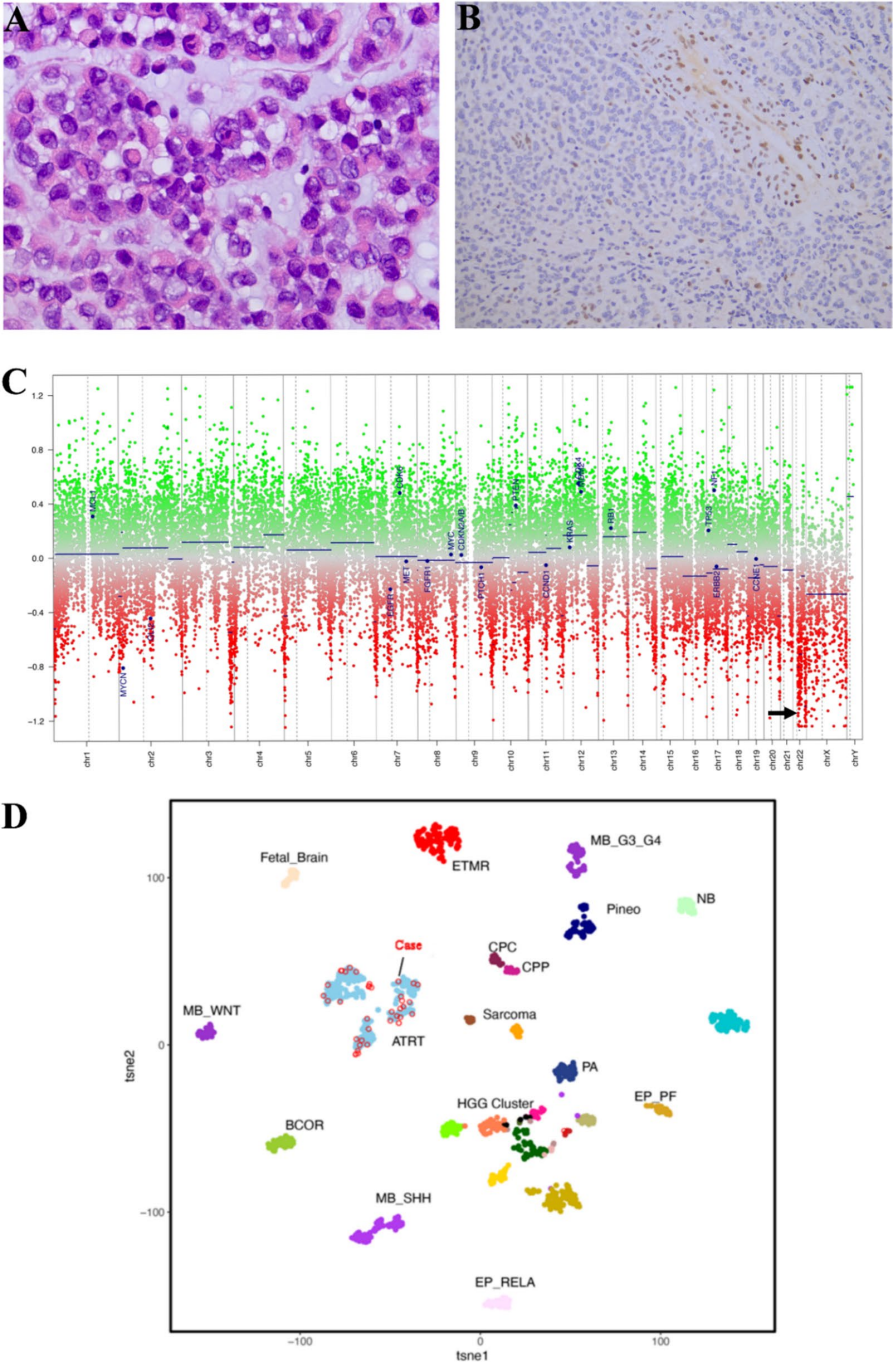
Morphology and molecular biology. Histological morphology reveals tumour rhabdoid cells arranged in sheets within a myxoid stroma, moderate eosinophilic cytoplasm, vesicular nuclei and pleomorphism (**A** 40 × magnification). Mitotic activity was high. Tumour cells revealed intranuclear loss of INI1 (**B** 20 × magnification). Copy number analysis (**C**) from the Illumina Epic Methylation array reveals loss of chromosome 22, housing SMARCB1 (black arrow). Subsequent unsupervised clustering of expanded paediatric tumour reference samples (*n* = 1200) against patient samples by *t*-distributed stochastic neighbour embedding (tSNE) dimensionality reduction analysis is shown (**D**). Individual samples are colour-coded in the respective tumour class. Red circle outlines indicate samples from the same batch of data

Histological examination diagnosed ATRT with highly cellular tumour infiltrating parenchyma (Fig. 2A). Tumour cells were positive for EMA but revealed intranuclear loss of INI1 (Fig. 2B). GFAP, NeuN and NFP were negative. The FISH analysis identified biallelic *SMARBC1* loss. Interrogation of the *SMARCB1* loss by next-generation sequencing identified a deletion upstream of exon 9 and a larger deletion (approximately 10 Mb) incorporating *SMARCB1* and extending upstream to *TBX* and downstream to *NIPSNAP1*. Methylation array profiling (Supplementary Information; Fig. 2C, D) confirmed both chromosome 22 loss and ATRT-MYC subgrouping. Results were verified against the MNP brain tumour classifier (calibration score 0.99).

**Fig. 3:**
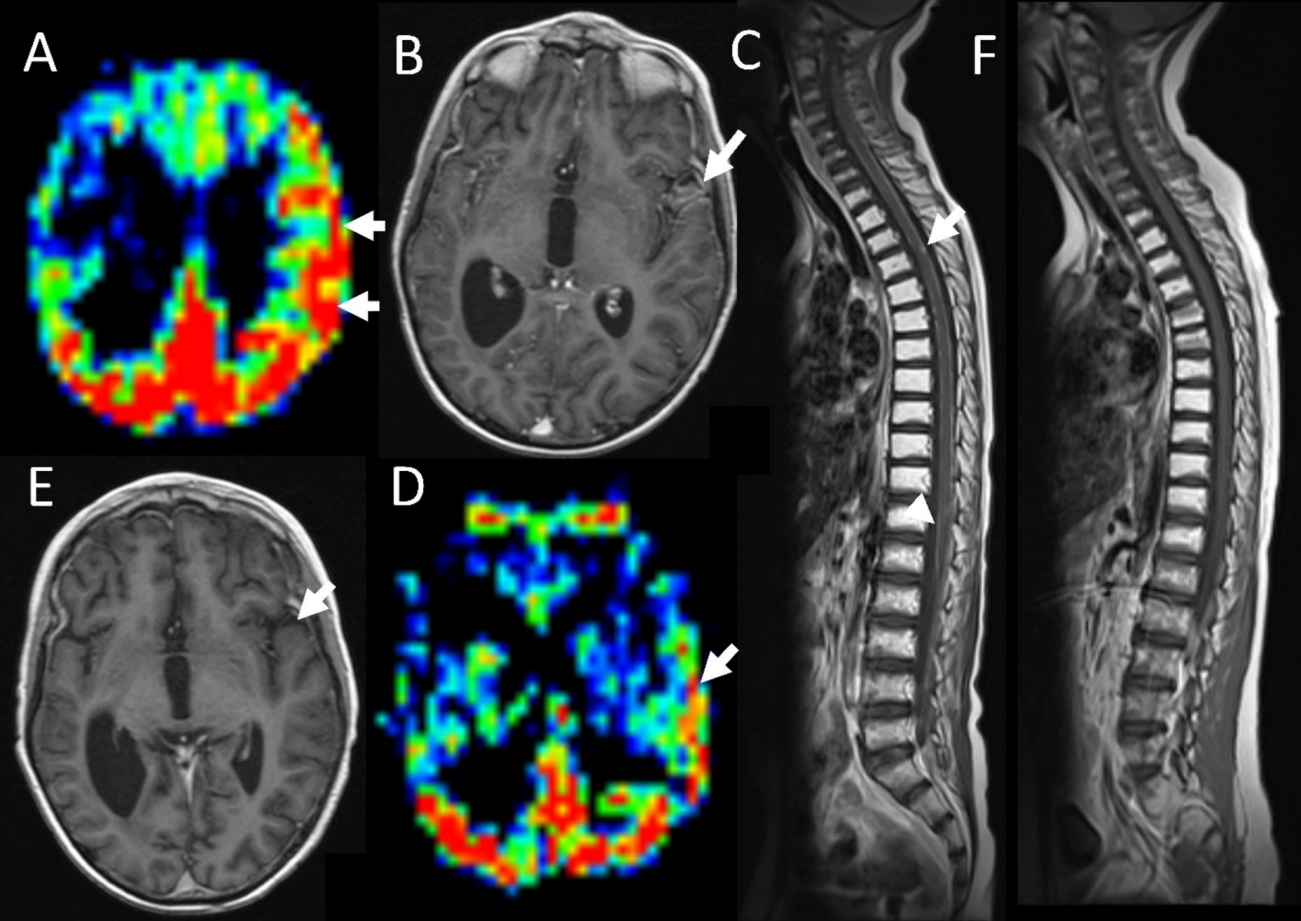
Presumed relapse and recovery imaging. At 4 months post-therapy, there was increasing ASL perfusion overlying the left cerebral hemisphere (panel A, white arrows). By 8 months, the axial T1 post-contrast imaging demonstrated increasing leptomeningeal enhancement in this distribution (panel B, white arrow). At the same timepoint, there was extensive spinal nodular disease (panel C, white arrow) as well as disease seen coating the whole of the spinal cord (panel C, white arrowhead). Following the cessation of therapy and initiation of palliative care, there was a significant improvement in imaging appearances. The leptomeningeal enhancement resolved (panel E, white arrow) and the degree of increased perfusion significantly improved (panel D, white arrow), although it never returned to baseline. The apparent spinal disease completely resolved (panel F)

The patient was treated with a combination of craniospinal radiotherapy (36 Gy neuraxial dose with 18 Gy boost to disease sites, in 30 fractions) set amongst EU-RHAB protocol chemotherapy incorporating pre-radiotherapy intraventricular methotrexate and systemic chemotherapy (doxorubicin × 3, ICE × 3, VCA × 3) [13] (Table 1). At the end of therapy, the patient was clinically stable with no convincing evidence of disease residuum. The previously seen enhancing leptomeningeal disease, diffusion restriction and increased perfusion changes had resolved (Fig. 1I–K). However, at 4 months post-therapy, imaging appearances had declined, initially with increasing perfusion overlying the left cortical mantle (Fig. 3A). This was gradually accompanied by corresponding increased diffusion restriction and leptomeningeal enhancement (Fig. 3B). By 8 months post-therapy, the patient had developed worsening of presumed neuraxial nodular and leptomeningeal disease coating the left cerebral hemisphere, spine and posterior fossa (Fig. 3C). This was accompanied clinically by intractable seizures,  reduced consciousness and deteriorating quality of life, despite oral etoposide and the EZH2 inhibitor, tazemetostat. Consequently, the patient embarked on a palliative pathway, incorporating only analgesia and several anti-seizure medications including sodium valproate. After a brief period of further clinical deterioration requiring escalation of supportive medication (including maximal sodium valproate dosing; 40 mg/kg/day), the patient unexpectedly began to improve with seizure control and improved consciousness, enabling a return to school. Repeat imaging 6 months following palliation showed resolution of almost all abnormalities in the brain and spine (Fig. 3D–F). Sadly, over the following months, the patient deteriorated again with recrudescence of both seizures and leptomeningeal disease on imaging, such that he succumbed to his disease 30 months from diagnosis and 1 year from commencing palliation.

## Discussion

Our patient is the longest survivor of PDL ATRT in the current literature (Table 1; [7–12]). The median survival of published cases is 8 months. Childhood cases are exceedingly rare; our patient is the fifth PDL ATRT reported. Indeed, one published case presented with a concomitant spinal mass lesion, questioning its inclusion. Besides our patient, only one other patient survived beyond 12 months from diagnosis. Adjuvant therapy for both longer-term survivors involved multimodal chemotherapy and high-dose craniospinal radiotherapy, suggesting a potential prognostic merit in application. However, such therapy is extremely difficult to maintain due to myelotoxicity and cannot be considered for the majority of young ATRT patients due to the deleterious neurocognitive impact such therapy has on the developing brain.

We are the first to use methylation profiling to assign MYC-subgroup status to a PDL ATRT. It could be considered unsurprising for this specific patient, given the propensity for this subgroup to arise in older ATRT patients, and its association with radiological peri-tumoural oedema, compared with the TYR and SHH subgroups [4]. Nevertheless, it would be of interest to verify if this subgroup is enriched in ATRT PDL irrespective of age.

Our case also utilised MRI ASL perfusion imaging to suggest an underlying malignant process as the responsible aetiology for this patient’s complex presentation. Modern multi-parametric techniques, including diffusion and perfusion-weighted imaging, provide supplementary information on tumour physiology and biology compared with conventional MRI [14]. These techniques are increasingly utilised as non-invasive adjuncts for improving the pre-operative classification of paediatric brain tumours [15]. We have shown that such sequences can also support a diagnostic MRI scan to affect a patient’s management, as well as provide useful follow-up imaging data. In this instance, the diagnostic diffusion sequences alone were misleading, but correlation with ASL perfusion imaging completed the radiological phenotype to suggest malignancy.

Our PDL ATRT patient demonstrated temporary clinical recovery during palliation from a radiologically diagnosed metastatic relapse, accompanied by imaging resolution of all suspected sites of tumour dissemination. The likeliest explanation is of pseudoprogression from radiation necrosis post craniospinal radiotherapy, which duly resolved before true relapse occurred. Differentiating radio-necrosis from tumour progression is an ongoing challenge in neuro-oncology and has caused misdiagnoses of relapse in other paediatric embryonal CNS tumours treated similarly [16, 17]. However, given there was gradually increased ASL perfusion and diffusion restriction, tissue enhancement infilling the subarachnoid cortical spaces, extra-axial intradural spinal nodularity as well as leptomeningeal enhancement coating the entire spine, such appearances were atypical for a classical, intra-axial, radiation necrosis phenotype. Tissue confirmation of relapse is clearly recommended in these scenarios. In our case, the patient was never deemed fit enough to undergo such surgery.

A less favoured hypothesis was that prior to discernible improvement, the patient had been commenced on sodium valproate, a histone de-acetylase inhibitor (HDACi) [18]. Histone deacetylases utilise chromatin remodelling to control genomic transcription [19]. In vitro work has described SWI/SNF interactions with HDACs in addition to  HDAC1 overexpression in rhabdoid cell lines [20–22], while HDAC inhibitors including valproate have been shown to diminish proliferation, restore cell cycle arrest and promote differentiation of ATRT cell lines [18, 23, 24]. Early phase studies of sodium valproate’s use as an HDACi in paediatric brain tumours have been published [25, 26], supporting further evaluation of HDACi as a potential therapy in ATRT. Further enthusiasm must be tempered since our patient initially demonstrated deterioration despite escalating doses of sodium valproate, while death from disease progression occurred despite maximal valproate dosing.

In conclusion, we present a rare case of paediatric MYC-subgroup PDL ATRT, using perfusion MR perfusion imaging, within a multi-parametric diagnostic package, as a marker for malignancy against other aetiologies. We administered intensive multimodal therapy, including high-dose craniospinal radiotherapy, resulting in the longest overall survival reported. An unanticipated clinical and radiological improvement following a radiological diagnosis of relapse highlights the importance of confirming recurrence by tissue extraction where feasible.

## Supplementary Information

Below is the link to the electronic supplementary material.

Supplementary file1 (DOCX 15 KB)

## Data Availability

This manuscript does not report data generation or analysis.

## Declarations

### Third party material

All of the material is owned by the authors and/or no permissions are required.
