# Supplementary material for: Primary diffuse leptomeningeal atypical teratoid/rhabdoid tumours (ATRT) of childhood: a molecularly characterised case report and literature review
Source: Childs Nerv Syst. 2025 Jan 22;41(1):93. doi: 10.1007/s00381-024-06698-w (PMC11754338; doi:10.1007/s00381-024-06698-w)
Supplement: Supplementary file 1 — Supplementary file1 (DOCX 15 KB) [file 381_2024_6698_MOESM1_ESM.docx]

**Primary diffuse leptomeningeal atypical teratoid/rhabdoid tumor (ATRT) of childhood; a molecularly characterized case report and literature review.**

**Supplementary Information:**

**Imaging:**

Imaging studies were acquired on the same 1.5T MRI Siemens Magnetom Avanto scanner (Siemens, Oxon UK). Standard brain tumor MRI sequences included (T2, T1 pre and post contrast T1 and FLAIR) plus supplementary physiological imaging parameters including diffusion weighted imaging (b value = 1000) and perfusion imaging. Perfusion was assessed by arterial spin labelling (ASL). ASL used pulsed PICORE labelling with QUIPSS II modification 23 and echo-planar imaging read-out. Scan parameters: inversion time (TI1) 0.7 seconds, TI2 1.5 seconds, TR 2.2 s, TE 16 ms and 45 pairs of label and control images.

**Molecular biology analysis:**

Genome-wide DNA methylation profiling of all samples was performed using the Infinium Methylation EPIC (EPIC) BeadChip (Illumina, San Diego, CA, USA) according to the manufacturer’s instructions.

Copy number analysis from the Illumina Epic Methylation array was generated using Conumee (Hovestadt V, Zapatka M. Conumee: Enhanced copy-number variation analysis using Illumina DNA methylation arrays. R package version 1.9.0, <http://bioconductor.org/packages/conumee/>).

Classification results (and calibration scores) from the Illumina Epic Methylation array were performed using the raw methylation array idat files, which were uploaded to the DNA methylation‐based classifier, MNP version 12.5 ([www.molecularneuropathology.org](http://www.molecularneuropathology.org); Heidelberg, Germany). The case’s methylation profile was established by comparing against a reference dataset of over 1,200 CNS tumours, and a pure ATRT reference dataset (n = 427) (Rare Brain Tumour Consortium, HSC, Toronto) using hierarchical clustering, non-negative factorization, t-distributed stochastic neighbor embedding and Uniform Manifold Approximation and Projection across 5 sets of representative probes. Results were verified against the MNP brain tumor classifier as defined above (Calibration score 0.99).
